# Intraoperative diagnosis with abnormal branching of the left A8 pulmonary artery from the left main pulmonary artery

**DOI:** 10.1186/s40792-018-0475-7

**Published:** 2018-07-03

**Authors:** Yasushi Mizukami, Nobuhito Ueda, Hirofumi Adachi

**Affiliations:** Department of Thoracic Surgery, National Hospital Organization, Hokkaido Cancer Center, 2-3-54 Kikusui 4-jo, Shiroishi-ku, Sapporo-shi, Hokkaido 003-0804 Japan

**Keywords:** Left pulmonary abnormal branch, Mediastinal basal pulmonary artery to S8, Lung cancer

## Abstract

**Background:**

Safety is of vital importance for lung resection. The dissection of pulmonary vessels is associated with vascular injury and bleeding, and identification of the vessels is necessary. The most common abnormal branching pattern of the left pulmonary artery is the mediastinal lingular artery. However, a mediastinal basal pulmonary artery is very rare. A case of abnormal branching from the left pulmonary artery to S8 which was diagnosed intraoperatively, and, thus, its dissection was avoided, is reported.

**Case presentation:**

A 76-year-old woman with rheumatoid arthritis was diagnosed with left upper lung adenocarcinoma and visited our hospital. Contrast CT was not performed due to renal dysfunction, and abnormal branching of the left pulmonary artery was not identified. Video-assisted thoracoscopic left upper lobectomy and lymphadenectomy were performed. After the upper pulmonary vein was dissected and tissue around it was detached carefully, a pulmonary mediastinal branch from the left main pulmonary artery was identified descending between the upper pulmonary vein and upper bronchus. It was possible to separate the interlobar fissure safely and preserve A8. On retrospective examination, non-contrast CT showed A8.

**Conclusions:**

Although preoperative identification of left pulmonary mediastinal branches was difficult by non-contrast CT, a careful surgical procedure preserved the left pulmonary mediastinal A8.

## Background

In lung resection, safety is vitally important. In particular, dissection of pulmonary vessels is associated with vascular injury and bleeding, and identification of the vessels is necessary. The most common abnormal branching pattern of the left pulmonary artery is the mediastinal lingular artery. However, a mediastinal basal pulmonary artery is very rare. A case of abnormal branching from the left pulmonary artery to S8 that was diagnosed intraoperatively and its dissection was avoided is reported.

## Case presentation

**Fig. 1:**
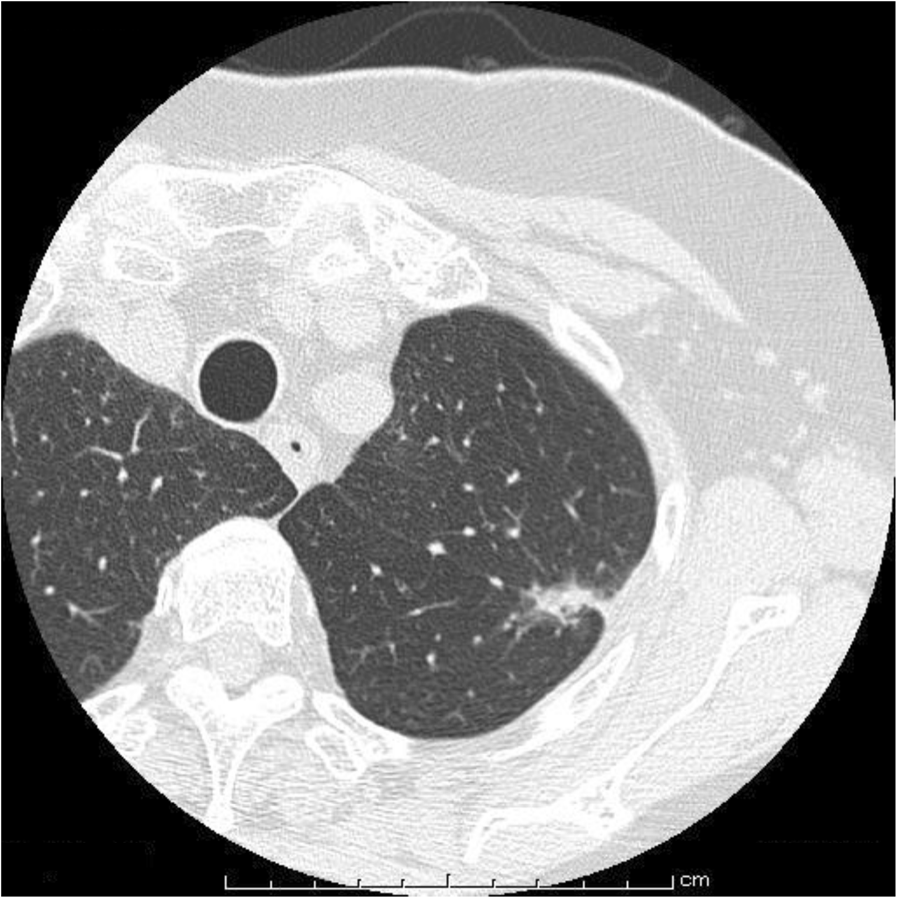
Chest-computed tomography findings. It shows the nodule of left S1+2 with indentation. It is 22 mm in size

**Fig. 2:**
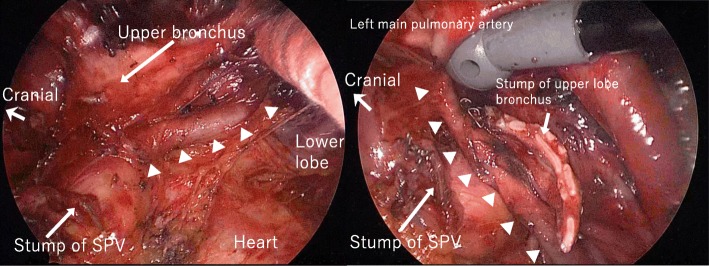
Intraoperative findings. Pulmonary artery branch from the left main pulmonary artery to S8 is between the stump of the superior pulmonary vein (SPV) and the upper lobe bronchus (white arrowheads)

**Fig. 3:**
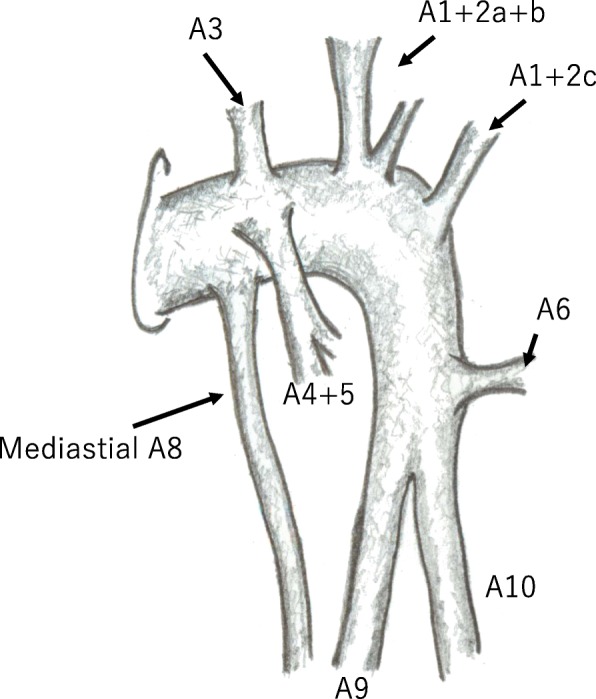
A schema of left pulmonary artery and branches. Left main pulmonary artery gives off mediastinal A4+5 and A8. Basal pulmonary artery supplies S9 and S10

**Fig. 4:**
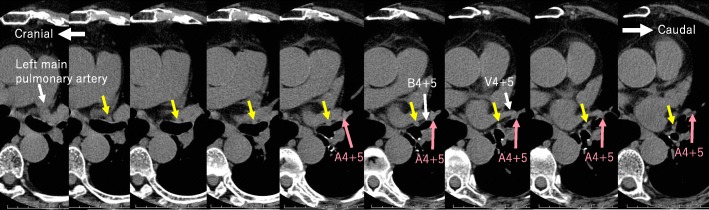
Chest-computed tomography (mediastinal window setting). It shows a pulmonary branch from the left main pulmonary artery to S8 (yellow arrow). It is different from mediastinal A4+5 (pink arrow). It is between the left superior pulmonary vein and the left upper lobe bronchus

The patient was a 76-year-old woman with rheumatoid arthritis. Computed tomography (CT) had been performed for a medical checkup, and a small nodule was detected in the left upper lobe (S1+2) a year before she visited our hospital. Follow-up CT showed that the nodule with indentation was growing to 22 mm in size, and she was referred to a nearby hospital (Fig. 1). Fluorodeoxyglucose (FDG)-positron emission tomography (PET), brain magnetic resonance imaging, and transbronchial biopsy were performed, showing left upper lung adenocarcinoma classified as cT1bN0M0 Stage IA according to the Union for International Cancer Control classification (seventh edition). Though contrast-enhanced three-dimensional computed tomographic angiography (3DCT) of the pulmonary vessels was usually performed to identify the pulmonary branches preoperatively at that time, it was not performed in the present case due to renal dysfunction, and abnormal branching of the left pulmonary artery was not identified. Video-assisted thoracoscopic left upper lobectomy and lymphadenectomy were performed. A1+2c, the upper pulmonary vein, mediastinal A4+5, A3, and A1+2a+b were detached carefully and dissected. Before separation of the interlobar fissure was completed, a pulmonary mediastinal branch from the left main pulmonary artery was identified descending between the upper pulmonary vein and upper bronchus. The interlobar fissure was separated safely with preservation of A8 (Figs. 2 and 3). On retrospective examination, non-contrast CT showed A8 (Fig. 4).

## Conclusions

An abnormal pulmonary branch can induce unexpected bleeding, and careful surgery is needed to dissect the branch to the preserved lobe. The number of left pulmonary branches varies from two to seven, and variations may occur in all segments. Generally, the first anterior branch supplies the lingular division in less than 10% of cases [1]. Recently, lingular artery branching patterns have been reported, with mediastinal origin in 9.2%, interlobar and mediastinal origin in 26.9%, and interlobar origin in 63.9%. Furthermore, 8 of 23 cases with mediastinal lingular artery were overlooked intraoperatively [2]. However, a branch from the first left pulmonary branch to the basal segment is rare.

It is possible that even the operator is not able to recognize a mediastinal lingular artery. Therefore, the possibility of missing a mediastinal basal lung artery is high. A total of 14 cases on the left side were reported only in Japan, including the present case (Table 1) [2–15].

**Table 1:** Abnormal branch of the left pulmonary artery to the lower lobe: a review of the literature

Report		First author	Year	Preoperative modality	Preoperative diagnosis
1	A9+10	Bamba	1985	Angiography	○
2	A5+8	Iwabuchi	1995	Contrast CT	×
3	A9+10	Sano	1996	Contrast CT	×
4	A8+9	Moriyama	2009	Contrast CT	○
5	A5+8+9+10	Kataoka	2010	Contrast CT	○
6	A8	Sueda	2011	Contrast CT	○
7	A9	Kaneda	2012	Contrast CT	×
8	A5+A8b	Kozu	2012	Contrast CT	×
9	A8+9b	Matsumoto	2012	3DCT	○
10	A8	Kato	2014	3DCT	×
11	A4+5+9+10	Yajima	2014	Non-contrast CT	×
12	A8b+9b+10	Kawai	2015	Contrast CT	○
13	A8+9+10	Sonoda	2016	3DCT	○
14	A5+8+10	Nagata	2016	3DCT	○
15	A8	Our case	2018	Non-contrast CT	×

It is possible to identify a basal lung artery preoperatively by contrast-enhanced CT or 3DCT. However, it has been reported that it was not diagnosed by non-contrast CT. In the present case, the mediastinal basal lung artery was identified intraoperatively, though it was difficult to diagnose it preoperatively. Therefore, the discreet separation of the vessel and bronchus is very important. Retrospectively, CT showed the mediastinal basal artery, although the interpretation was difficult; careful CT reading is also important.

**Fig. 5:**
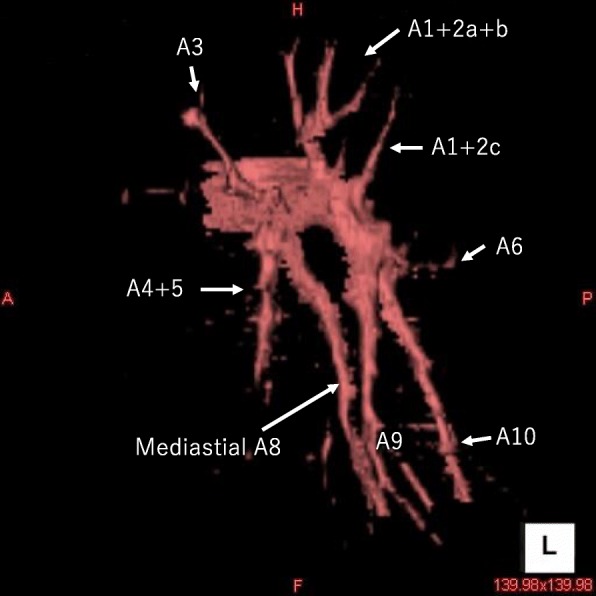
Three-dimensional computed tomographic pulmonary angiography of our case without contrast medium. It was constructed by non-contrast CT retrospectively. It is easy to recognize left mediastinal A8 given off from main pulmonary artery

3DCT is useful to identify the running condition of the pulmonary vessels preoperatively. However, contrast medium should be avoided when there is renal dysfunction, allergy, and so on. On the other hand, 3DCT without contrast medium has been reported to have slightly poorer resolution but may be helpful [16]. Actually, we constructed 3DCT of our case using non-contrast CT retrospectively (Fig. 5). It was necessary to spend several hours to make this image because it was difficult to distinguish artery from vein without contrast medium. The surface of pulmonary artery may be jagged; however, it helps to comprehend pulmonary pattern of branching.

In conclusion, although preoperative identification of left pulmonary mediastinal branches was difficult on non-contrast CT, a careful surgical procedure preserved the left pulmonary mediastinal A8.

## Abbreviations

3DCT: Three-dimensional computed tomographic angiography
CT: Computed tomography
FDG-PET: Fluorodeoxyglucose-positron emission tomography
